# Gegen Qinlian Decoction Ameliorates Nonalcoholic Fatty Liver Disease in Rats *via* Oxidative Stress, Inflammation, and the NLRP3 Signal Axis

**DOI:** 10.1155/2021/6659445

**Published:** 2021-02-16

**Authors:** Yuqi Ying, Haitao Zhang, Dian Yu, Wei Zhang, Dongling Zhou, Shuangchun Liu

**Affiliations:** ^1^College of Second Clinical Medicine, Zhejiang Chinese Medical University, Hangzhou 310053, Zhejiang, China; ^2^Department of Urinary Surgery, Taizhou Municipal Hospital, Taizhou 318000, Zhejiang, China; ^3^Department of Endocrinology, Taizhou Municipal Hospital, Taizhou, Zhejiang, China; ^4^Department of Nursing, Taizhou Municipal Hospital, Taizhou, Zhejiang, China; ^5^Department of Blood Transfusion Division, Taizhou Municipal Hospital, Taizhou, Zhejiang, China

## Abstract

Gegen Qinlian Decoction (GQD), a classic Chinese herbal formula, has been widely used in Chinese clinic for centuries and is well defined in treating nonalcoholic fatty liver disease (NAFLD). However, the mechanism action of GQD on NAFLD is still rarely evaluated. The present study aims to investigate the effect of GQD on treatment of NAFLD in rats and to further explore the underlying mechanism. The rat NAFLD model established by high-fat-diet feeding was used in the research. Our results exhibited the liver lesions and steatosis was significantly alleviated in NAFLD rats treated with GQD via Oil Red O and H&E staining. Body weight and liver index in GQD groups were reduced significantly (*P* < 0.05). Moreover, the biochemical analyzer test results showed that GQD significantly decreased blood lipid levels total cholesterol (TC), triglycerides (TG), low-density lipoprotein cholesterol (LDL-C), and liver injury indicators alanine aminotransferase (ALT), aspartate aminotransferase (AST), and alkaline phosphatase (ALP), while it increased the level of high-density lipoprotein cholesterol (HDL-C) (*P* < 0.05). The levels of interferon-*β* (IFN-*β*), tumor necrosis factor-*α* (TNF-*α*), and malondialdehyde (MDA) after the GQD treatment were significantly lower, and then interleukin-2 (IL-2), superoxide dismutase (SOD), and glutathione peroxidase (GSH-Px) levels were lifted significantly (*P* < 0.05). Further, GQD blocked the expression of NLRP3, ASC, caspase-1 mRNA, and proteins in the liver tissues significantly (*P* < 0.05). These findings indicated that GQD can ameliorate the hepatic steatosis and injury of NAFLD. Its possible mechanism involves the modulation of inflammatory cytokines and antioxidative stress and the inhibition of NLRP3 signal axis activation. The results support that GQD may be a promising candidate in the treatment of NAFLD.

## 1. Introduction

Nonalcoholic fatty liver disease (NAFLD) refers to the main clinical manifestation of pathological syndrome caused by excessive liver fat deposition rather than the definite factor drinking [1, 2]. Its progress period includes simple steatosis, nonalcoholic steatohepatitis (NASH), fibrosis, and cirrhosis [3, 4]. Nowadays, NAFLD has become one of the common liver diseases, the course of NAFLD varies with the progress, and the main pathological features are steatosis and fat storage [5]. However, there are still not enough safety and effective therapeutic agents so far, and seeking NAFLD potential and promising drugs is always the current focus of attention [6].

Natural herb products are the abundant resources and have been given special attention for NAFLD drug development [2, 7]. Traditional Chinese Medicine (TCM) has been used clinically since ancient China, and most of TCM compound formulas are composed of herbs. Several TCM formulas or extracts are considered as potential candidates in treating NAFLD [2, 7–9]. As is known, the pathological mechanisms of NAFLD are complicated and incompletely understood, and the action of the TCM formulas to anti-NAFLD is usually unclear. TCM theories consider most complications of NAFLD as Yang deficiency and phlegm-dampness; accordingly, correcting the deficiency and moving the phlegm are effective strategies in treating the disease [10, 11]. Gegen Qinlian Decoction (GQD), a TCM classical formulation, is derived from the classical legend of TCM, “Treatise on Cold Pathogenic Diseases” (*Shanghan Lun* in Chinese), written by Zhongjing Zhang in Han Dynasty [12]. It is composed of four TCM herbs: Gegen (*Puerariae Lobatae Radix*, from *Pueraria lobata* (Willd.) Ohwi (Fabaceae)), Huangqin (*Scutellariae Radix*, from *Scutellaria baicalensis* Georgi (Lamiaceae)), Huanglian (*Coptidis Rhizoma* from *Coptis chinensis* (Ranunculaceae)), and Zhigancao (*Glycyrrhizae Radix et Rhizoma Praeparata cum Melle* from *Glycyrrhiza uralensis* (Fabaceae)) [13]. GQD has had the effect of clearing away heat and promoting health and relieving muscle and dispersing evil in clinic for thousands of years in East Asia, which matches the strategy described above [14, 15]. In modern laboratories findings, GQD exerts the function of lowering blood glucose, blood lipid, anti-inflammation, antioxidation, and so forth [16–19]; meanwhile, as reported, GQD can improve nonalcoholic steatohepatitis in rats by resisting insulin and blood lipid or via gut microbiota [20, 21]. However, the NAFLD therapeutic overall efficacy and molecular mechanism remain to be further evaluated.

It is reported that an important type of receptor NLRP3 inflammatory signal axis exists in innate immunity, named, NLRP3-ASC-caspase-1 signal axis (NLRP3 signal axis) [22, 23], which can mediate NAFLD-induced inflammation and oxidative stress response [24, 25]. In the present study, we used high-fat-induced NAFLD rats to evaluate the effect of TCM intervention. The paper intends to determine the levels of serum lipids, inflammatory factors, and antioxidant factors of Gegen Qinlian Decoction (GQD) in NAFLD rats and further investigate its regulatory effect on NLRP3 signal axis to explore the potential mechanism of GQD intervention in rat NAFLD.

## 2. Materials and Methods

### 2.1. Chemicals and Reagents

The four Chinese herbs of GQD were purchased from Chinese Herbal Medicine Co., Ltd. of Zhejiang Chinese Medicine University (Lot: 20181213, 20160523, 20160718, and 20181025, respectively) and certified by Professor Zhang Shuili, an Associate Professor at the School of Pharmacy, Zhejiang Chinese Medicine University (Hangzhou, China), in addition to atorvastatin (Lot: PZ0001, Sigma-Aldrich, USA), rat triglyceride (TG), low-density lipoprotein cholesterol (LDL-C) kit (Lot: 20180324 and 20180330, Shanghai Fusheng Industrial Co., Ltd. China), TRIzol Reagent (Lot: 20150502, Invitrogen Co., USA), SYBR Green PCR and Reverse Transcription kit (Lot: A5001-1, AA406-1; TaKaRa Co., Japan), rabbit anti-rat primary antibodies NLRP3, ASC, and caspase-1 (Lot: 08063965, 2813878, and GR202191-1; Abcam Co., UK), rabbit anti-rat GAPDH primary antibody, horseradish peroxidase- (HRP-) labeled goat anti-rabbit secondary antibody IgG, and protein molecular mass marker and BCA protein quantification kit (Lot: 00021408, 00081307, 00131456, and 23072018; Kangwei Century Biotechnology Co., Ltd., Beijing, China).

### 2.2. Preparation of GQD

GQD extracts were prepared according to the method of the Chinese Pharmacopoeia [13]. The herbal material Pueraria Lobatae Radix (250 g) was soaked in 2 L of cold water for 30 min before being boiled for 30 min alone. The other herbs, including Scutellariae Radix (150 g), Coptidis Rhizoma (150 g), and Glycyrrhiza Radix (100 g), were added and boiled together with Pueraria Lobatae Radix for 60 min. The first decoction was thus obtained. The mixture was boiled a second time with an additional 1.5 L of water for 30 min to obtain the second decoction. Finally, the first and second decoctions were mixed, filtered through gauze, and concentrated to a final extract in a weight ratio of 5 : 3:3 : 2 [20]. Extensive chemical studies have mentioned that flavones (free form and glycosides), flavanones, alkaloids, and triterpene saponins are the major compounds in GQD, especially the active ingredients, baicalin, and berberine hydrochloride [26].

### 2.3. Animal Model and Experimental Design

Specific Pathogen-Free (SPF) male Sprague Dawley (SD) rats, weighing 180 ± 20 g, were supplied from the Animal Experimental Center, Zhejiang Academy of Medical Sciences, China [Certificate no. SCXK (Zhe) 2018-0033]. The rats were housed in a circumstance with controlled temperature (20∼25°C) and humidity (40∼45%) and a 12 : 12 h light/dark cycle. Rats were supplied with granular food and could access water freely as normal rodents. The experiments were conducted in accordance with local guidelines for experimental animal care in Zhejiang Chinese Medicine University, which were approved by the Institutional Research Ethical Committee for the use of laboratory animals.

After SD rats were fed adaptively in the environment of 12 h and 26°C for 7 days, they were randomly divided into a control group, a model group, an atorvastatin group, and GQD high-, medium-, and low-dose group, with 10 rats in each group. According to the previous reports [20, 27, 28] and preliminary experiments, the high, medium, and low doses of GQD were determined to be 8, 4, and 2 g/kg/d (about 1, 2, and 4 times of human dose, respectively), and atorvastatin was 2.0 mg/kg/d. Except that the control group uses common feed, the model group and each treatment group are fed with high-fat diet (ratio of fat : protein : carbohydrate is 3 : 1 : 1) for nonalcoholic fatty liver modeling. After 8 weeks, gavage treatment was performed according to the above dose, with a gavage volume of 2.0 mL/100 g; the control group and the model group were given 0.9% saline in equal volume and gavage once a day for 4 weeks. Animals were weighed every week. They were fasted for 12 h before the last dose and anesthetized with pentobarbital sodium, blood was taken from the abdominal aorta, and serum was centrifuged; the rats were sacrificed and dissected to collect the liver.

### 2.4. Histopathological Section Observation by Oil Red O Staining

The liver tissues of rats were collected and washed in normal saline at 4°C, and the surface water was blotted dry with filter paper and then immediately put into 4% formalin solution for fixation. After 1 week, the specimens were embedded in paraffin and regular sections (thickness 4 *μ*m); then the slices were placed in the Oil Red O and hematoxylin & eosin (H&E) staining solution and rinsed with distilled water for 30 min, and finally the slides were sealed. All the samples were examined and observed under an optical microscope.

### 2.5. Serum Biochemical Analysis

A Hitachi automatic biochemical analyzer (Wako, Richmond, VA, USA) was used to detect serum lipid levels in rats total cholesterol (TC), triglycerides (TG), low-density lipoprotein cholesterol (LDL-C), and high-density lipoprotein cholesterol (HDL-C), as well as liver injury indicators alanine aminotransferase (ALT), aspartate aminotransferase (AST), and alkaline phosphatase (ALP). Meanwhile, ELISA assay was used for serum inflammatory cytokines interleukin-2 (IL-2), interferon-*β* (IFN-*β*), tumor necrosis factor-*α* (TNF-*α*), and antioxidant makers superoxide dismutase (SOD), glutathione peroxidase (GSH-Px), and malondialdehyde (MDA) expression (Lot: 20170655IL2M, 20175200IFNM, 20170052TNFM, 20176020NOM, 20174899SODM, and 20146660GSHM, respectively; R&D Co., USA).

### 2.6. Real-Time Quantitative PCR Assay to Test the Key Target Genes of NLRP3 Signaling Axis in Liver Tissue

The tissues around the rat liver were removed and homogenized quickly. The total RNA was extracted according to the instructions of the RNA extraction kit; then the total RNA concentration and absorbance A were measured. Each RNA sample was reversely transcribed as cDNA through the reverse transcription kit. The above cDNA was subjected to PCR amplification under the following conditions: predenaturation at 95°C for 10 min, denaturation at 95°C for 15 s, annealing at 60°C, and extension for 45 s, for a total of 40 cycles. GAPDH was used as an internal reference gene to detect NLRP3, ASC, and caspase-1 gene expression on a real-time PCR detector. The Ct value of each sample is obtained by instrument analysis, and all sample genes are normalized by the internal reference GAPDH, and the 2^*−*△△Ct^ method is used to calculate mRNA relative expression. △△Ct is given in [29]. The primers were synthesized by Shanghai Bioengineering Co., Ltd., and the primer sequences are shown in Table 1.

### 2.7. Western Blotting Assay to Determine the NLRP3 Signaling Axis Key Target Proteins in Liver Tissue

Liver tissues were quickly homogenized; then RIPA lysate was added at a ratio of 1 : 10 (g/ml), and the total protein content was measured according to the instructions of the BCA protein quantification kit, to ensure that the sample volume is the same. After the protein of each group was lysated and extracted, it was separated by SDS-PAGE electrophoresis and transferred to nitrocellulose membrane, and the converted PVDF membrane was blocked in 5% skim milk powder solution at room temperature for 2 h, and TBST washing solution was rinsed for 3∼4 times. Then the following procedure was launched: Add primary antibody and incubate overnight at 4°C. Rinse 3 to 4 times, add HRP-labeled secondary antibody, incubate at room temperature for 2 h, and rinse 3 to 4 times. Chemiluminescence detection substrate ECL working solution was used for color development. ImageJ analysis system was used to analyze the protein western blot in grayscale, and the protein expression levels of NLRP3, ASC, and caspase-1 in the liver tissue from each group were tested and compared.

### 2.8. Statistical Analysis

The experimental data were processed by SPSS 17.0 software. ELISA data analysis was carried out using ELISA Calc regression/fitting computing software, and the results were expressed as the mean ± standard deviation (‾x ± s). The two-tailed Student's *t*-test was used to compare two groups and one-way ANOVA with Tukey's post hoc test was performed for the comparison among multiple groups. *P* < 0.05 indicated that the difference was significant.

## 3. Results

### 3.1. Effects of GQD on NAFLD in Rats

**Figure 1:**
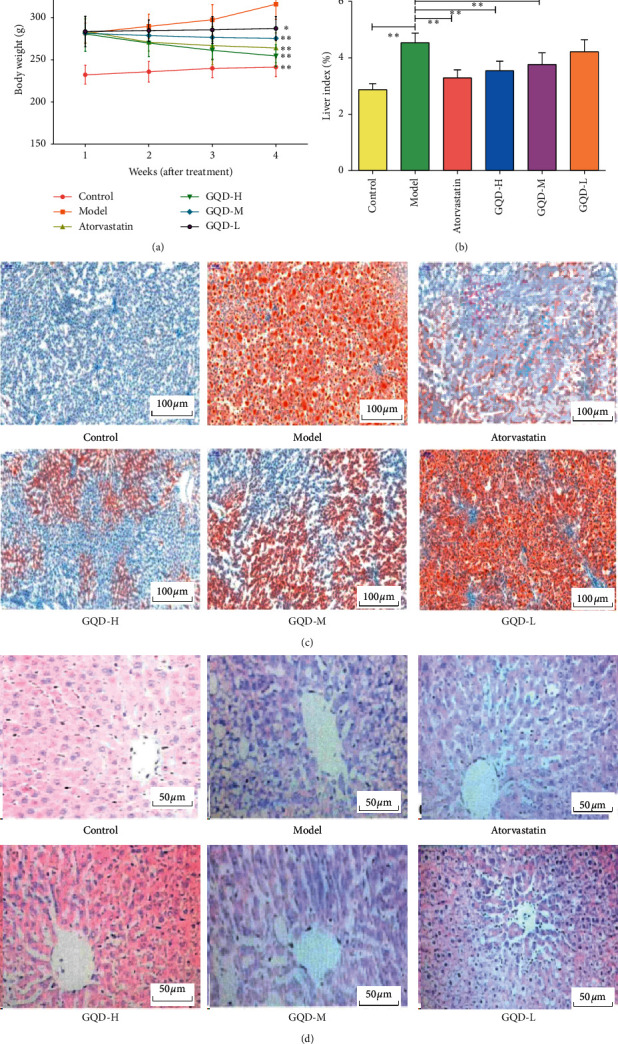
GQD alleviated the NAFLD in rats. (a) Effect on body weight. (b) Effect on liver index. (c) Histological section observation of liver tissues by Oil Red O staining (magnification x200). (d) Histopathological examination of liver tissues by H&E staining (magnification ×400).

To evaluate the effects of GQD on treatment of NAFLD rats, the body weight and liver index were monitored. As shown in A and B in Figure 1, the body weight and liver index of NAFLD rats after GQD intervention showed a significant mitigation compared with model group (*P* < 0.05). In addition, this effect among the high, medium, and low doses of GQD group reflected a dose-effect relationship. Thereby, the low dose of GQD group seemed to exhibit the intervention but not obviously.

The Oil Red O and H&E staining results showed that the liver slices of the control group showed no obvious lesions and no lipid droplets. Compared with the control group, the model group had significant obvious increases in steatosis and lipid droplets, with noticeable hepatic steatosis and infiltration of inflammatory cells in the centrilobular and portal areas, which also confirmed the success in establishing the nonalcoholic fatty liver model. The GQD groups had improved lipid droplets and lesions in different degrees compared with the model group. From pathological slices, it could be seen that the GQD high-dose group showed the best improvement, close to atorvastatin. It shows that GQD can improve the lipid storage of rat liver caused by high-fat diet and reduce hepatic steatosis. The results are shown in Figures 1(c) and 1(d).

### 3.2. Effects of GQD on Serum Biochemical Parameters in NAFLD Rats

**Figure 2:**
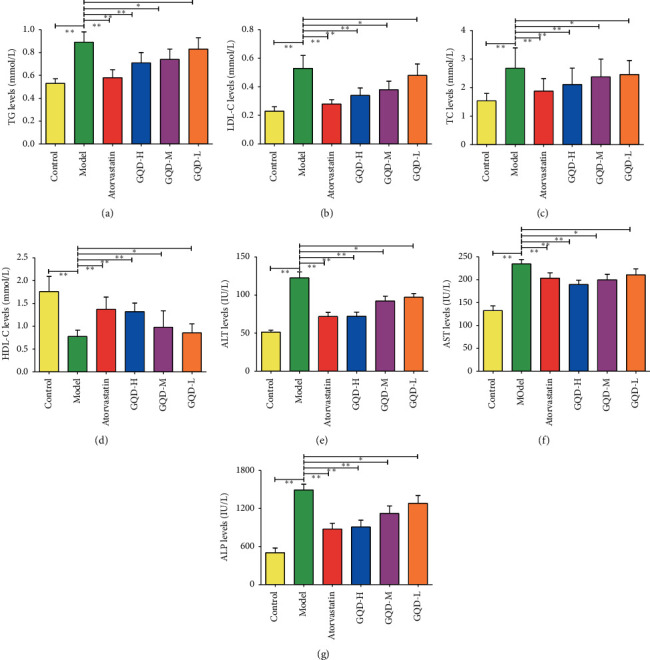
The effect of GQD on blood lipid related indexes in NAFLD rats (*n* = 10) ((a) TG, (b) LDL-C, (c) TC, (d) HDL-C, (e) ALT, (f) AST, and (g) ALP). Note: compared with the model group,  ^*∗*^*P* < 0.05; ^*∗∗*^*P* < 0.01.

GQD showed a favorable therapeutic efficacy in NAFLD; in order to investigate the regulation of NAFLD-related biomolecules in rats, the serum TG, LDL-C, TC, HDL-C, ALT, AST, and ALP were detected. The results showed that the blood lipid expressions of TG, TG, and LDL-C were increased in the model group, while HDL-C was significantly decreased. The liver injury indicators levels ALT, AST, and ALP in the model group were significantly increased compared with the control group, respectively (*P*˂0.05). The GQD high-, medium-, low-dose group in blood lipid levels, compared with the model group, had significantly decreased TG, TG, and LDL-C levels, while HDL-C was increased (*P* < 0.05). Moreover, the liver injury indicators levels ALT, AST, and ALP in GQD groups were decreased significantly (*P* < 0.05). These showed that GQD can modulate the metabolism of blood lipid and liver injury indicators to ameliorate NAFLD in rats (Figure 2).

### 3.3. GQD Mediated Inflammatory Cytokines and Antioxidant Markers

**Figure 3:**
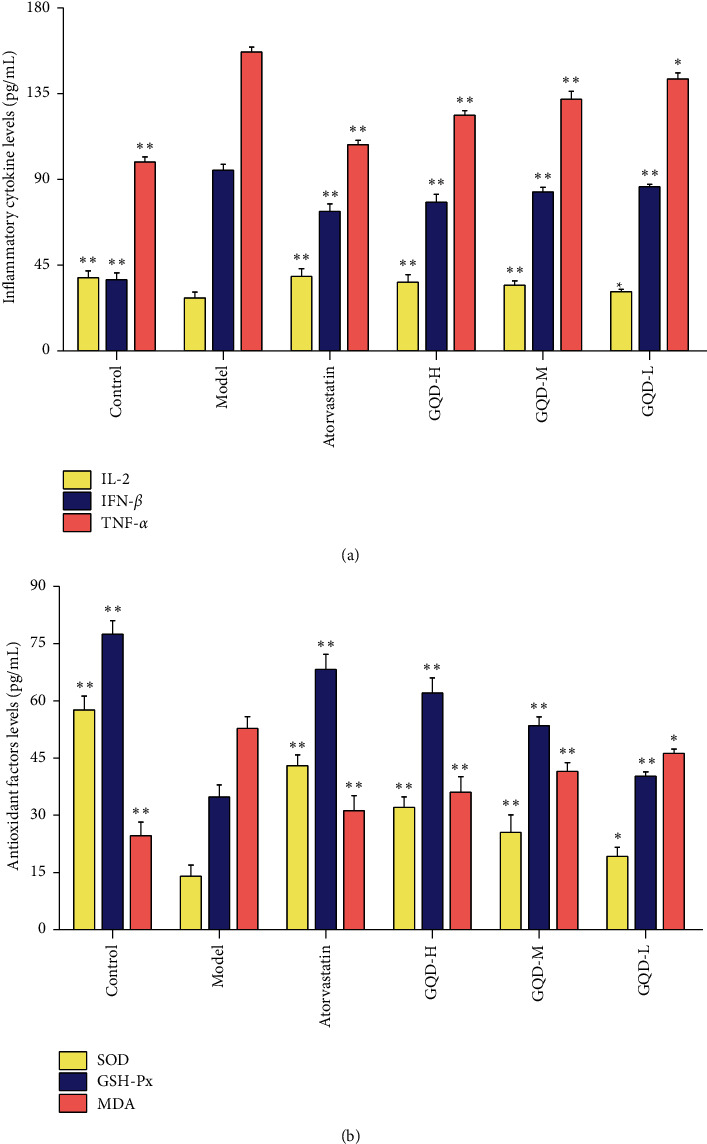
Effects of GQD on serum inflammatory cytokines and antioxidant factors in NAFLD rats (*n* = 10). (a) Regulation of GQD on rat serum IL-2, IFN-*β*, and TNF-*α* levels. (b) Effects of GQD on SOD, GSH-Px, and MDA secretion. Note: compared with the model group,  ^*∗*^*P* < 0.05: ^*∗∗*^*P* < 0.01.

To explore the modulation in inflammation and oxidative stress of the GQD on NAFLD, cytokines and antioxidant markers levels test was performed (Figure 3). Compared with the control group, the levels of IFN-*β*, TNF-*α*, and MDA in the model group were significantly increased, while the levels of IL-2, SOD, and GSH-Px were significantly reduced (*P* < 0.01). The GQD groups lowered the expression of IFN-*β*, TNF-*α*, and MDA in rats to different degrees and the expressions of IL-2, SOD, and GSH-Px were increased (*P* < 0.05); and the high-dose group had the strongest regulation effect. It indicated that GQD could suppress the expression levels of IFN-*β*, TNF-*α*, and MDA in rat serum and increase the expression levels of IL-2, SOD, and GSH-Px, suggesting that GQD can modulate the inflammation and block the oxidative stress in NAFLD rats.

### 3.4. GQD Inhibited the NLRP3 Signaling Axis of Liver Tissue in NAFLD Rats

**Figure 4:**
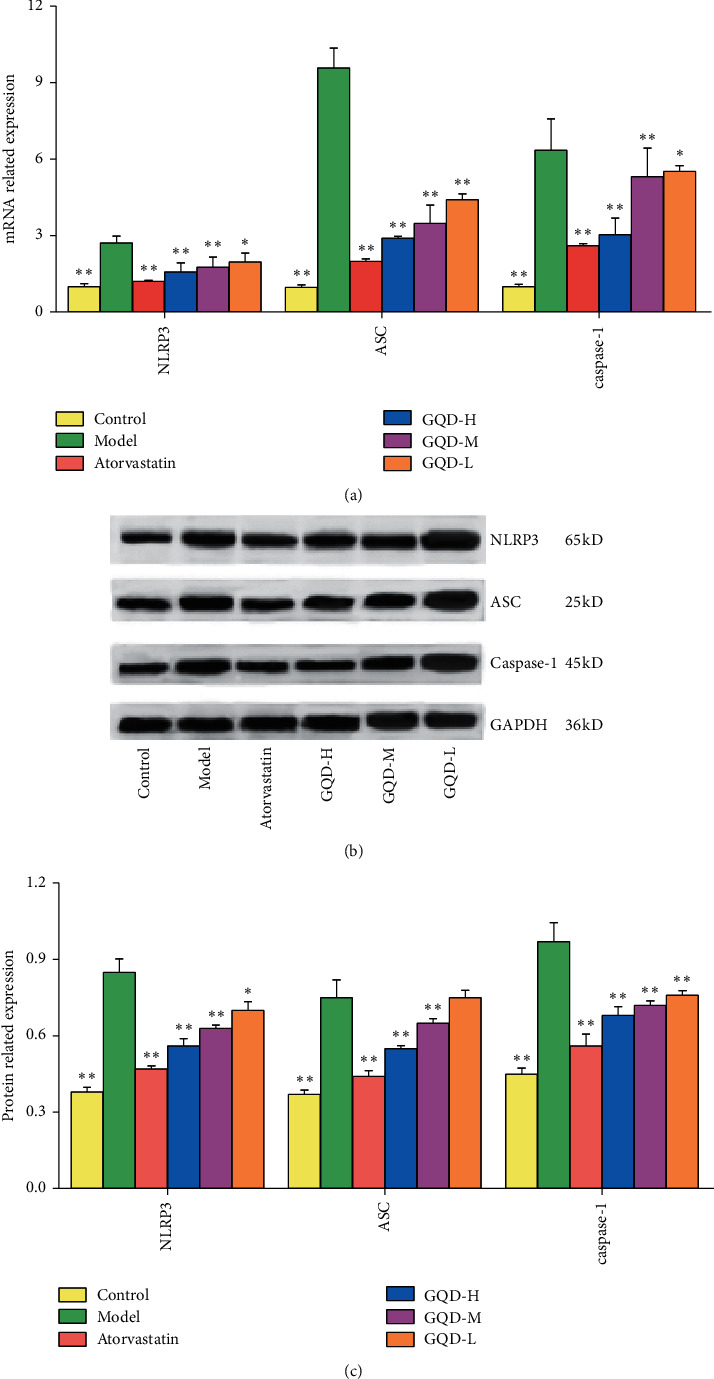
GQD inhibited the NLRP3 signal axis key targets in NAFLD rat liver tissue. (a) Effect of GQD on the NLRP3 signal axis mRNA in liver tissue of NAFLD rat. (b) Western blot graph. (c) Effect of GQD on the NLRP3 signal axis proteins in liver tissue of NAFLD rat. Note: compared with the model group,  ^*∗*^*P* < 0.05; ^*∗∗*^*P* < 0.01.

GQD exposure for 4 weeks significantly suppressed the inflammation and oxidative stress, which could be mediated by NLRP3 inflammasome as previously reported [24]. Meanwhile the involvement of NLRP3 inflammasome related signal axis activation in NAFLD progression was clearly shown [24, 25]. In comparison with control, the expression levels of NLRP3, ASC, caspase-1 protein, and mRNA in liver tissue from the model group were significantly increased (*P* < 0.01); compared with the model group, the expression levels of NLRP3, ASC, caspase-1 protein, and mRNA were significantly reduced (except the expression of ASC protein in the low-dose group of GQD, all *P* < 0.05); the expression level of each GQD group was higher than that of atorvastatin group (shown in Figure 4 and Supplementary Figure S1). It showed that GQD can inhibit the activation of NLRP3/ASC/caspase-1 signal axis.

## 4. Discussion

The pathogenesis of nonalcoholic fatty liver disease (NAFLD) is complex, involved with hepatic parenchymal cells, nonparenchymal cells, immune cells, and multiple cytokines or pathways, and often further develops into more serious diseases such as cirrhosis and liver cancer [30, 31]. Those key targets of pathophysiological mechanisms are important for the development of noninvasive markers of NASH and liver fibrosis as well as targeted therapeutic drugs. The present clinical treatment mainly aims at single target and pathway, and the curative effect is not satisfactory. Therefore, the development of safe and effective drugs, especially drugs combined with multiple factors and links in the progression of NASH and liver fibrosis, has become a new strategy for the treatment of NAFLD [32, 33]. Traditional Chinese Medicine (TCM) is known for its characteristics of multiple pathways and multiple targets, and TCM has relatively few side effects in the treatment of NAFLD [34]. As reported, GQD alleviates nonalcoholic steatohepatitis associated liver injuries via anti-inflammatory response and inhibition of toll-like receptor 4 signaling pathways [20]. In the present study, the TCM prescription Gegen Qinlian Decoction (GQD) was taken to study its intervention effect on NAFLD in rats. Regarding this, it is generally believed that the progression of hepatic fibrosis can be used as one of the most important endpoints to judge the efficacy of drugs [32]. The pathological analysis results showed that GQD could significantly relieve liver disease and lipid degeneration in rats and block the NAFLD progression. The results of histopathology indicated that GQD exhibited a promising mitigation on fatty disorders of NAFLD.

The prestage of NAFLD is mainly the accumulation of lipid droplets, mainly due to the excessive accumulation of TC, TG, LDL-C, and other lipids in serum and/or liver, and the decrease of HDL-C, which usually occurs when a large number of lipids influx and exceed liver lipid clearance [35, 36]. Moreover, AST, ALT, and ALP are common indicators of liver metabolism and transport function, and once liver function is impaired, its serum level can be significantly increased [37, 38]. The World Health Organization (WHO) considers ALT as the most sensitive indicator of liver damage [39]. In this regard, the levels of related blood lipid TG, LDL-C, TC, and HDL-C and liver injury indicators ALT, AST, and ALP in rats were investigated. The results found that the levels of TG, LDL-C, TC, ALT, AST, and ALP were significantly reduced, while HDL-C was increased after GQD treatment. It can be seen that GQD can mediate the normal metabolism of blood lipids and decrease the liver injury to inhibit NAFLD lesions and prevent their development.

Nucleotide binding oligomerization domain-like receptor protein 3 (NLRP3) is a class of cytoplasmic pattern recognition receptors [40]. Yang et al. reported that NLRP3 signal axis exerted a vital role on NAFLD in a mouse model induced by high-fat diet [40]. Thereinto, caspase-1 is an active product of NLRP3 signal axis, with its adapter protein ASC and procaspase-1, which consist of NLRP3. Studies have shown that NLRP3 knockout in nonalcoholic steatohepatitis (NASH) mice model inhibited the activation of NLRP3, ASC, and caspase-1 in liver and can significantly reduce liver inflammation in mice and prevent the development of the NASH disease [40, 41]. Besides, reports have shown that the NLRP3 signal axis is involved in the regulation of important inflammatory cytokines such as IL-6 and IFN-*β* [42–44] and antioxidant factors such as MDA, SOD, and GSH-Px [25, 45]. Animals fed with HFD has been reported to induce obesity, hyper/dyslipidemia, hepatic steatosis, oxidative stress, mild fibrosis, and enhanced level of cytokines. Thus, amelioration of oxidative stress and inflammation cytokines could be regarded as an anti-NAFLD viable mechanism. Collectively, as this regard, blocking the NLRP3 signal axis to antioxidant and anti-inflammation is a strategy in treating NAFLD rat model induced by a high-fat diet. Due to the accumulation of triglycerides and free fatty acids in NAFLD rat, excessive reactive oxygen species (ROS) are generated in the liver. The free radical metabolism balance in the body, including SOD and GSH-Px, is mainly maintained by the antioxidant system [46]. MDA is the main product of lipid peroxide degradation, which not only prevents mitochondrial respiratory chain electron transfer but also promotes inflammatory response, thus promoting liver antioxidant enzyme activity (SOD, CAT, and GSH-Px) and inhibiting peroxidation products MDA content and antioxidative stress can promote lipid metabolism and prevent NAFLD caused by obesity [25, 47].

Based on those, while studying the regulation of GQD on inflammation and oxidative stress, we further investigated the expression of the NLRP3 signal axis to explore the mechanism of GQD improving NAFLD in rats. The results show that GQD can significantly decrease the serum levels of IFN-*β*, TNF-*α*, and MDA in NAFLD rats and increase the expressions of IL-2, SOD, and GSH-Px. GQD can significantly inhibit the expression levels of NLRP3, ASC, and caspase-1 genes and proteins in liver tissue and block the activation of NLRP3 signal axis. It is suggested that GQD can regulate inflammatory cytokines, inhibit the level of oxidative stress, and downregulate the expressions of key genes and proteins of the NLRP3 signal axis. It has been proved that the above possible anti-NAFLD mechanism strategy is feasible. However, as a multicomponent and multitarget drug, GQD is specific to which component or multiple components playing the effect on NAFLD, and the relevance of the mechanism needs to be deepened.

In conclusion, this study examines the mechanism of GQD treatment of NAFLD from the inflammatory cytokines, oxidative stress, and NLRP3 signal axis. From the animal, molecular, and protein levels, it is initially confirmed that GQD can inhibit NLRP3 signal axis by regulating inflammatory cytokines and oxidative stress. The study well established that GQD improves NAFLD lesions and provides a reference for the research and clinical application of GQD in the treatment of NAFLD. The findings provided scientific evidence in support of GQD being used as one kind of folk remedy to treat NAFLD in China. Further, we will explore how GQD treats NAFLD in connection to the NLRP3 signal axis with inflammation and oxidative stress.

## Figures and Tables

**Table 1:** Real-time fluorescence quantitative PCR primer sequence.

Gene	Primer sequences		Primer length
NLRP3	Forward	5' CTCGCATTGGTTCTGAGCTCA 3'	153 bp
	Reverse	5' AGTAAGGCCGGAATTCACCA 3'	
ASC	Forward	5' TGGAGTCGTATGGCTTGGAG3'	300 bp
	Reverse	5' TGTCCTTCAGTCAGCACACT3'	
Caspase-1	Forward	5' ACTCGTACACGTCTTGCCCTC 3'	190 bp
	Reverse	5' CTGGGCAGGCAGCAAATTC 3'	
GAPDH	Forward	5' GTGACACCCACTCTTCCACC 3'	162 bp
	Reverse	5' GTGGTCCAGGAGGCTCTTAC 3'	

## Data Availability

The datasets used and/or analyzed during the current study are available from the corresponding author upon reasonable request.

## Supplementary Materials

Supplementary Materials Supplementary Figure S1: the original western blots of the NLRP3 signal axis proteins in liver tissue of NAFLD rat. Supplementary Figure S2: graphical abstract.
