# Supplementary material for: Gegen Qinlian Decoction Ameliorates Nonalcoholic Fatty Liver Disease in Rats via Oxidative Stress, Inflammation, and the NLRP3 Signal Axis
Source: Evid Based Complement Alternat Med. 2021 Feb 16;2021:6659445. doi: 10.1155/2021/6659445 (PMC7902151; doi:10.1155/2021/6659445)

# Graphical abstract

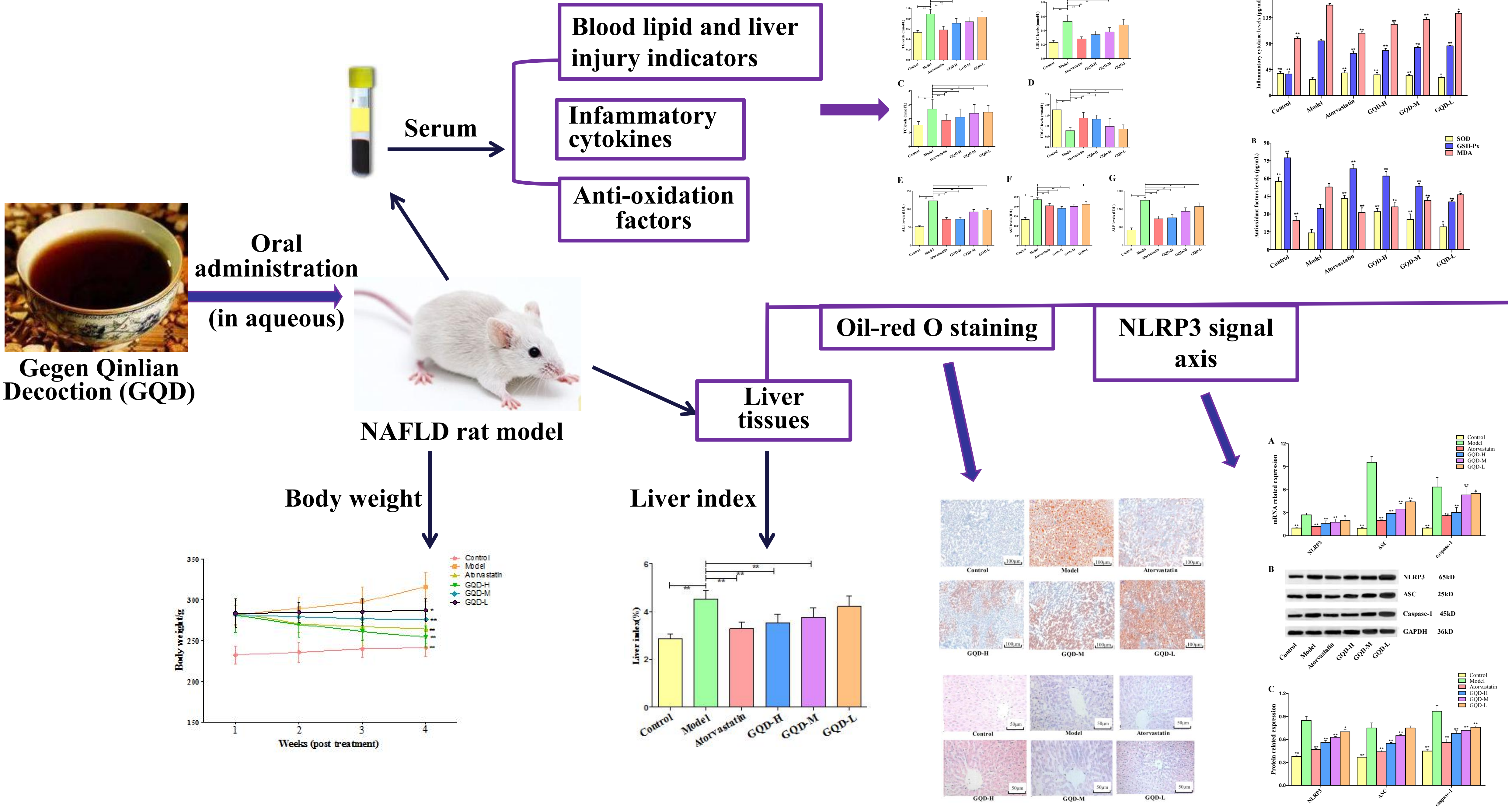

Supplement: Supplementary Materials — Supplementary Figure S1: the original western blots of the NLRP3 signal axis proteins in liver tissue of NAFLD rat. Supplementary Figure S2: graphical abstract. [file 6659445.f1.zip › 6659445.f1/Graphical abstract (1).pdf]
